# Global Perspectives in Acute Kidney Injury: Spain

**DOI:** 10.34067/KID.0000000000000080

**Published:** 2023-02-15

**Authors:** María José Soler, Angel Luis Martin de Francisco, Natalia Ramos

**Affiliations:** 1 Department of Nephrology, Vall d’Hebron Hospital Universitari, Vall d’Hebron Barcelona Hospital Campus, Nephrology and Transplantation Group, Vall d’Hebron Institut de Recerca (VHIR), Passeig Vall d'Hebron, Barcelona, Spain; 2 Department of Nephrology, Hospital Universitario Valdecilla, Universidad de Cantabria, Santander, Cantabria, Spain

**Keywords:** acute kidney injury and ICU nephrology, acute kidney injury, renal replacement therapy, Spain

## Introduction

Although AKI is a global public health problem, there are few data on the global epidemiology in Spain. This lack of information has also been observed in other countries and may be in part related to the fact that this disease can be diagnosed and observed in different settings, such as intensive care units, emergency departments, and hospitalization facilities involving several medical departments. AKI has a clear effect on health cost related to an increase in hospital stay that has been demonstrated to have a negative effect in morbimortality.^1^

In Spain, nephrology departments are mainly reserved to third-level hospitals, most of which are university level. This confers a great heterogeneity in the treatment of AKI, given the fact that in most second-level hospitals, there are no nephrologists, and, therefore, it is managed by internists and intensivists. In the current perspective, we summarized AKI in Spain for epidemiology and organizational structures for its proper diagnosis, treatment, and prevention.

## Epidemiology of AKI

There are few data on the global epidemiology of AKI in Spain, thus information for promoting and implementing strategies for early diagnosis and prevention is needed. This is the main reason, among others, why the working group on AKI has been recently created under the umbrella of the Spanish Society of Nephrology but without an AKI national registry yet. In 1996, Liaño *et al.* in the framework of the Madrid AKI Study Group prospectively studied the incidence, etiology, and clinical features of AKI in patients older than 14 years admitted during a 9-month period by any of the 13 tertiary care hospitals in Madrid, Spain, with a population of approximately four million at that time. AKI was considered when a sudden rise in serum creatinine concentration of more than 2 mg/dl was found in patients with normal renal function or when a sudden rise of 50% or higher was observed in patients with previous chronic renal failure. The incidence of AKI in Madrid was a total of 209 cases per million population (p.m.p) distributed in acute tubular necrosis, 88 cases p.m.p; prerenal, 46 p.m.p, AKI on chronic kidney disease, 29 p.m.p; obstructive, 23 p.m.p; and others. A later study published in 2021 in a single tertiary hospital in Madrid found within a 2-year period an incidence of 12.3% (6629/54,095) of community-acquired AKI (CA-AKI).^2^ These data are higher than the other study that was between 5.9% (11,546/192,435) and 6.3% (3298/49,971).^3^ Although we do not have recent epidemiological studies, the incidence of hospital-acquired AKI (HA-AKI) is similar according to a previous study published by Safadi *et al.*^4^ In addition, in 2006, a study named FRAMI (*Fracaso Renal Agudo en Medicina Intensiva*) focused on the epidemiology of AKI in the Spanish Intensive Care Units led by the Spanish working group of current status of acute renal failure and techniques from Spanish Society of Critical Intensive Medicine and Coronary Units was published. They performed a prospective study of adult patients admitted during 8 months in 43 Spanish intensive care units (ICU) with AKI defined as creatinine ≥2 mg/dl or diuresis<400 ml/24 hours (chronic patients 100% increase of creatinine, excluding those with baseline creatinine ≥4 mg/dl). There was a total of 901 episodes of AKI (incidence 5.7%), 55% of which occurred on admission. Of these, 38.4% of the episodes were due to acute tubular necrosis, 36.6% to prerenal, and 21.2% to mixed. HA-AKI was associated with abdominal surgery, cardiovascular surgery, and urological surgery, followed by congestive heart failure, hemiplegia/paraplegia, and urgent admission.^2^ In Spain, absolute decrease in plasma volume (prerenal AKI) and drug-induced AKI have been identified as the main causes of HA-AKI.^2,5^ We have no data about causes of community-acquired AKI, which are probably similar to another previous studies with similar risk factors as for HA-AKI.^6^ Renal replacement therapy (RRT) was required in 38%. It should be noted that most of the patients were men (70.6%) with a mean age of 60.7 years. As expected, patients with AKI in ICU had a higher mortality rate (46.8%) than patients without AKI (14.2%) suggesting that AKI worsens the prognosis of ICU patients.^7^ Later studies focused on the AKI epidemiology have been mainly performed by cardiologists in the context of cardiac surgery, heart failure, or atrial fibrillation, among others (5.5%–10.2%).^7^ In addition, the COVID-19 pandemic has driven the study of AKI associated with COVID-19 in Spain that is in part related to the direct role of the virus on the ACE2 tubular receptors that are the main binding site of SARS-CoV-2.^8^ Portoles *et al.* described that 21% of patients with COVID-19 admitted at hospital had an increase of creatinine; of them, 43.5% had previous CKD. In addition, the authors also described an 11.4% incidence of HA-AKI in patients with COVID-19 with normal previous serum creatinine levels. These patients had an increase of mortality similar to other causes of AKI.^9^ Taking all of these results with the few data that we have until now, it seems that in Spain the incidence of CA-AKI is similar to HA-AKI.

## Organizational Structures of Hospitals and ICU

Unfortunately, in Spain as in other countries, there is a great variability in the care for critically ill patients with AKI, with large variations across institutions, as the field lacks consensus on how and when to start RRT in ICU patients. Many institutions have less-than-ideal collaborations between nephrologists and intensivists, some programs fail to offer adequate educational training of nurses, and only a few of them have incorporated quality improvement programs designed to maximize the effectiveness and minimize complications of RRT delivery.^10^

In Spain, there are high-quality public and private hospitals. In most of them, mainly university centers, there are developed nephrology-driven services that admit and treat patients with AKI. In addition, most of our ICU are opened and have a designated nephrologist who works side-by-side with the intensivist to take care of patients with AKI. Together they decide which ultrafiltration objective every patient need or in intermittent hemodialysis (IHD). Nephrologists prescribe the HD and decide frequency and type of RRT with the intention of ensuring that the objectives settled with ICU doctors are achieved. Normally, the tertiary hospitals have on-site 24-hour on call nephrologists who are the referents for AKI in emergency departments. In hospitals where the treatment with CRRT depends on the nephrology department, it is prescribed by a nephrologist in agreement with the intensivist during 24 hours, including weekends. This practice is not homogeneous and varies between hospitals. The number of nephrologists dedicated to ICU is variable and depends on the type of relationship, number of nephrologists in nephrology department, etc. As nephrologists, we are in favor of this system because it ensures the early diagnosis, prevention, and treatment of the patient with AKI which is crucial in the first hours of the episode. Furthermore, it is already known that AKI episodes, although nonsevere, increase the risk of developing CKD,^11^ indicating again that if the nephrologist is involved in the AKI episodes perhaps CKD may be prevented, rapidly diagnosed, and treated if needed.

The AKI group of the Spanish Society of Nephrology has proposed some objectives for the nephrologist at the ICU which are summarized in (Table 1): (1) Presence and dedication for evaluation of etiology of AKI and decision making in nutrition, fluid overload, and supportive treatment; (2) coordination of research projects with proposals, such as epidemiological studies, biomarkers of early damage, preventive strategies; (3) promotion of continual medical education at different levels (students, nurses, ICU doctors, hospital staff…); and (4) follow-up of renal outcome after AKI.^12^

**Table 1:** Limitations and proposed solutions for improving AKI management in Spain

Limitations	Proposed Solutions
• Real AKI incidence unknown	• To create a national AKI registry
• Lack of consensus in the approach and treatment	• Standardization of treatment through consensus document
• Unknown of the type of renal replacement therapies used	• To carry out questionnaires in different hospitals
• Recognized lack of specialization within nephrology/dialysis nursing	• Promote nursing specialization in a regulated manner
• Few research groups focused on AKI	• Promote research through the AKI group of the Spanish Society of Nephrology • Implement the use of artificial intelligence for the treatment of big data.

## Methodology of Diagnosis of AKI and Prophylactic Prevention of AKI in High-Risk Patients

Currently, in Spain, the use of AKIN, RIFLE, or KDIGO recommendations has been indistinctly used for AKI diagnosis, with the preference for the KDIGO definition or AKIN classification in most published studies.^3,9,13^ In the daily clinical practice, all the different nephrology departments use the creatinine level, and in a few of them, the determination of cystatin is used in particular cases, but its use has not been widely expanded. The limited use of cystatin is related to the lack of availability in all of the hospitals and the delay in obtaining the results, for that reason it is not useful for early AKI screening and diagnosis. Biomarkers of cell cycle arrest as insulin-like growth factor-binding protein 7 (IGFBP7) and tissue inhibitor of metalloproteinases-2 (TIMP-2) are nowadays used in clinical and basic research.^14,15^

The use of risk prediction models and equations for AKI has not been homogeneously implemented in the Spanish hospitals; however, at the clinical research level, some Spanish studies have been performed.^2,8^ Martin-Cleary *et al.* generated a prediction model for HA-AKI named Madrid Acute Kidney Injury Prediction Score (MAKIPS). This score has an AUC-ROC of 0.81. This model is an online tool (http://www.bioestadistica.net/MAKIPS.aspx) that can be automatically calculated from electronic clinical records to predict the risk of HA-AKI at admission. They suggest that with this online tool the prediction of AKI risk may be more useful in decreasing the incidence of AKI than current electronic alerts, allowing the implementation of bundles cares.^2^ In this study, the external validation was performed at the same hospital. In parallel, another predictive model of HA-AKI in noncritically ill patients with data from Hospital del Vall d'Hebron was developed with an AUC-ROC of 0.907. In this study by Segarra *et al.*, they integrated the information of six electronic health databases commonly used in clinical practice to develop and validate a predictive dynamic model that allows one to accurately estimate the individual likelihood of suffering AKI at any time during a hospital stay in noncritically ill patients. Two sets of risk factors were identified: the first one with the demographic data and the patient's chronic comorbidities and the second included a set of risk factors related to the patient's clinical status and to the exposure to major surgery or nephrotoxic drugs (including contrast media) during the hospital stay. This model as compared with previous ones permits estimating the risk of HA-AKI to patients admitted to noncritical hospital wards. In addition, because it has been performed with data from one of the biggest hospitals in Barcelona with the most clinically complex programs, such as cardiac surgery and bone marrow transplantation, this allows findings to be applied in other hospitals with different characteristics and complexities.^16^ Probably the register of nephrotoxic drugs and contrast media use and the number of exposures to them is one of the limitations of MAPIKS study, and, as we described above, these are some of the main causes of HA-AKI. This could also explain the fact that the predictive model of Segarra *et al.* has an AUC-ROC higher than previous studies.

The use of prophylactic measures employed in high-risk patients has not been standardized in a Spanish national consensus. Last consensus dated from 2007 (Guía de actuación del FRAcaso renal agudo, SEN), and nowadays, the new guidelines and Spanish consensus are under revision. Thus, each hospital has used their measures following the KDIGO recommendations. Initially, the prophylactic strategies have been mainly based on hydration, avoiding nephrotoxic drugs, and bicarbonate and n-acetylcysteine in patients with known CKD that were exposed to nephrotoxic drugs or substances such as ionized contrast. Later, in a seminal publication in the NEJM, the benefit of the use of n-acetylcysteine as a prophylaxis was clearly questioned, and the main benefit was ascribed to the hydration previous to the administration of the contrast agent.^17^ For that reason, the administration of n-acetylcysteine has been individualized and not standardized in our country, and hydration is considered as the only preventive measure.^18^

## RRT in AKI: Management, RRT Used, Availability, and Costs

In Spain, in tertiary hospitals, the nephrologist plays a relevant role in the management and treatment of AKI in ICU, and in some of them, the prescription of continuous therapy completely depends on the nephrology staff*.* However, this responsibility is not homogeneous. Who takes care of RRT in AKI depends on different factors: (1) the particularity of each center, (2) the need or not of ICU admission, (3) interpersonal relationships between intensivists and nephrologists, and (4) the person in charge of technical implementation (continuous kidney replacement) in hospitals without nephrology department; this technique depends on ICU, but is the only type RRT that they offer. For that reason, when AKI is not recovered, the patient must be transferred to a tertiary hospital with nephrology department. Interestingly, in the past decade, more and more multidisciplinary teams have been implemented for the treatment of these types of patients. As mentioned above, regarding hospitalization wards, a nephrology expertise is needed for severe AKI.

The different varieties of RRT are chosen according to availability and experience of each center involved. There is not enough evidence of any RRT modality having superior benefits for patient survival, length of intensive care unit/hospital stay, or renal outcomes among critically ill patients, in spite of optimization of clinical indication, modality, timing of initiation, and intensity of initial therapy. This is still a controverted matter because only early start CRRT seems to be beneficial over intermittent hemodialysis among hemodynamically unstable patients.^9^ For this issue and for lack of realistic data, an AKI group has been created in the Spanish Society of Nephrology. During the following year, the results of a poll conducted among nephrologists working in different Spanish hospitals will answer the reality of clinical practice in patients with AKI who require RRT in Spain.

Most critical patients start continuous renal replacement therapy (CRRT) as a first dialysis technique because of hemodynamic instability. In hospital clinic, CRRT was used as first choice therapy if the patient needs more than 0.1 *μ*g/kg per minute of noradrenalin by protocol.^13^ In tertiary hospitals, with nephrology support, it is possible to use of prolonged intermittent renal replacement therapy (PIRRT) as a bridge therapy. Currently, an observational study is being conducted to evaluate the real effect of PIRRT in Catalonia (EC/19/111/5512). Unfortunately, there are few data about the use of different types of RRT, and the latest guideline did not specify about criteria of the use of one or another.^12^ After CRRT implementation, peritoneal dialysis fell into discussion because of a good result in volume control. Almost 90% of ICUs have CRRT, regardless of whether they belong or not to the public health system. As previously mentioned, when an intermittent dialysis technique is required, a nephrology unit is necessary. Thus, in Spain, these patients are at that moment transferred to a tertiary hospital. Very few second-level hospitals have a nephrology unit. We must keep in mind that these are patients of great complexity and high mortality rates.

In relation to the use of different types of CRRT (continuous venovenous hemofiltration, continuous venovenous hemodiafiltration, continuous venovenous hemodialysis…) there is not enough evidence for any one; thus, therapy election depends on the expertise of each team. When nephrologists are implicated in the decision, the election depends on the aim of depuration and the etiology of AKI (fluid overload, sepsis AKI, rhabdomyolysis…). Unfortunately, we do not have AKI registers in Spain, so it is difficult to know the frequency of the different therapies and different complications in relation to catheter, coagulation, etc. Citrate, heparin, or no coagulation is widely used in different ICU units according to their own protocol and experience.

CRRT is more expensive than IHD for materials and nurse team; however, in our country we have a Universal National Health Service that covers care and costs for all patients. In a private system, it depends on contracted insurance, but if the private insurance does not finance all the assistance, the patient is transferred to a public service.

## Outcomes of AKI

Recently with the outbreak of COVID-19 disease, different studies were published focused in the incidence and mortality after AKI.^9,13^ Piñeiro *et al.* studied 237 patients with severe COVID-19 with ICU admission; they described 21.4% of patients with AKI ≥2. Fifteen patients (28.85%) needed RRT, most of them with CRRT (13 patients). The authors described that in 71.15% patients, the cause of AKI was related to severe COVID-19. As similar previous studies, mortality was higher in patients with AKI vs patients without AKI (51.92% versus 7.3%).^13^ Portoles *et al.* described HA-AKI of 11.4% (ICU and no ICU wards). In the same way, patients with AKI had increased rate of mortality.^9^ Advanced age and CKD, together with major surgery and nephrotoxic drugs, are the main risk factors for AKI. Carpio *et al.* designed a predictive model to identify patients at high risk for AKI.^14^ Interestingly, Acosta *et al.* described differences in outcomes referring AKI on patients with previous normal kidney function as compared with patients with CKD.^15^ The authors described two profiles of patients according to the type of AKI, pure AKI, or acute on CKD, and the latter have a high probability of remaining on dialysis on discharge (30%).

**Figure 1:**
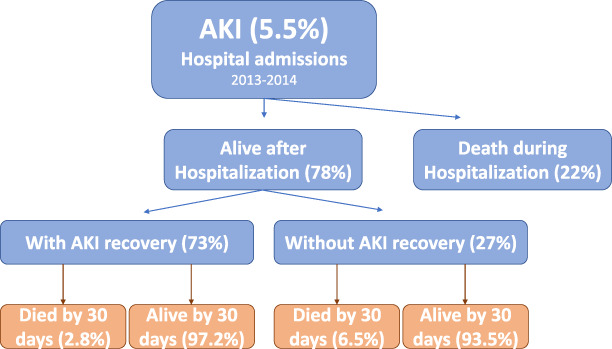
**Hospital-acquired AKI and survival in a single tertiary center in Spain.** Adapted from Quiroga *et al.*^20^

Survivors of AKI do not universally have a benign course. On long-term follow-up (1–10 years), approximately 12.5% of survivors of AKI are dialysis dependent; rates range widely, from 1% to 64%, depending on the patient population. From 19% to 31% of survivors experience partial recovery of kidney function and have chronic kidney disease^19^*.* In a retrospective study conducted by Quiroga *et al.*, they analyzed a total of 1194 patients with AKI alive after hospitalization admitted at tertiary hospital (Figure 1). The main cause of AKI was prerenal. Only 15% were AKI 3, and most (66%) were AKI 1. Seventy-three percent of patients recovered baseline kidney function, and 27% presented a persistent chronic kidney dysfunction. The predictor factors for nonkidney recovery were age, previous CKD, severity of AKI, heart failure history, admission blood pressure, and hemoglobin levels. The authors described an increased mortality at 30 days of discharge in the group with persistent chronic kidney dysfunction (6.5% versus 2.8%).^20^

Those survivors with severe AKI who required RRT are usually known by nephrology units and subsequently followed. However, a lot of patients get lost, and general practitioners are responsible of their follow-up, in many cases without knowing the history of a previous episode of AKI. This is possible because in some hospitals, AKI in ICU is managed exclusively by intensivists or a nephrology department is lacking. This disparity is in part related to the lack of protocol in the evaluation, management, and follow-up of this type of patients. To our knowledge, the differences for outcomes of severe AKI or AKI requiring RRT between second-level vs. academic university hospitals in Spain have not been previously studied. It is assumed that in our country, the implementation of multidisciplinary teams and a better diagnostic recorder and good follow-up protocols validated by the different medical societies (nephrology, intensivist, anesthesia, internal medicine and family, and community medicine) will improve long-term outcomes (see Table 1).

## Nephrology Education in Spain

The nephrology education in Spain is a four-year fellowship program that starts after the Internal Medical Resident (MIR) examination. It is a complete learning process where the fellows learn most of the knowledge needed to become future independent nephrologists. Around 100 nephrologists graduate in our country per year. Nephrology is a competitive specialty but not as well appreciated as cardiology for example. The reason for the previous statement seems in part to be related to less private clinical work and lack of extensive development of interventional nephrology. The training programs are mainly located or close to major cities. Once our young nephrologists finish their fellowship, they have work either in hospitals or in peripheral hemodialysis centers. In some hospitals, they combine in-hospital and peripheral hemodialysis centers, but usually their practice is focused in one of them. The nurses in Spain receive a special formation, but there is no special training like as MIR established, unified, and recognized like in other nurse subspecialties, such as midwives or pediatrics. Currently, there is no a conference of AKI in Spain. As previously mentioned, the AKI Group of the Spanish Society of Nephrology has been recently funded; for that reason it is expected that this type of conferences will be started in the next future.
